# Genetic and Clinical Features in *TP53*‐Mutated Patients With Myelodysplastic Neoplasms: A Retrospective Study Based on Next‐Generation Sequencing Data

**DOI:** 10.1002/cnr2.70584

**Published:** 2026-05-29

**Authors:** Yanming Cheng, Lixia Liu, Yu Gao, Yifei Wang, Wei Zhu, Hong Chen, Jiayue Qin, Yudi Zhang

**Affiliations:** ^1^ Zhejiang Provincial Key Laboratory of Pancreatic Disease The First Affiliated Hospital, Zhejiang University School of Medicine Hangzhou China; ^2^ Department of Medical Affairs Acornmed Biotechnology Co. Ltd. Beijing China; ^3^ Department of Hematology The First Affiliated Hospital, Zhejiang University School of Medicine Hangzhou China; ^4^ Myelodysplastic Syndromes Diagnosis and Therapy Center The First Affiliated Hospital, Zhejiang University School of Medicine Hangzhou China; ^5^ Cancer Center, Zhejiang University Hangzhou China; ^6^ Zhejiang Provincial Key Lab of Hematopoietic Malignancy, Zhejiang University Hangzhou China

**Keywords:** clinical features, MDS, NGS, *TP53* mutation

## Abstract

**Background:**

The recent fifth edition of World Health Organization and International Consensus Classification in 2022 recognized *TP53*‐mutated myelodysplastic neoplasms (MDS) as a distinct entity requiring multi‐hit classification. Literatures indicate that *TP53* variant alle frequency (VAF) correlates with clinical outcomes. However, comprehensive studies that characterize the full mutation landscape and clinical correlations in Asian populations are still limited.

**Aims:**

This research aims to elucidate the mutation spectrum and baseline characteristics of *TP53*‐mutated MDS in a Chinese cohort, thereby enhancing clinical understanding and treatment approaches.

**Methods and Results:**

A total of 161 *TP53*‐mutated patients from 1589 newly diagnosed MDS with next‐generation sequencing (NGS) data via a targeted 96‐gene sequencing panel were analyzed to assess genomic alterations. Among 161 *TP53*‐mutated patients (10.1% of 1589 MDS cases), we identified a predominance of missense mutations (76.9%) and frequent co‐occurrence with epigenetic modifiers, notably *DNMT3A* (11.8%), *TET2* (10.6%), and *ASXL1* (9.3%). Critically, *TP53* VAF correlated strongly with disease severity parameters, including hemoglobin levels, blast percentage, karyotype, and International Prognostic Scoring System (IPSS), revised IPSS (IPSS‐R) risk and IPSS‐Molecular (IPSS‐M) classifications (all *p* < 0.05), suggesting that *TP53* VAF serves as an important biomarker for risk stratification. In addition, patients with multiple *TP53* mutations harbored significantly worse IPSS, IPSS‐R, and IPSS‐M risk classifications compared to those with a single *TP53* mutation.

**Conclusions:**

This study underscores the importance of *TP53* mutations in Asian MDS patients, contributing to genetic profiling in risk stratification and therapeutic decision‐making.

## 1 Introduction

Myelodysplastic neoplasms (MDS) represent a heterogeneous group of clonal hematological disorders characterized by ineffective hematopoiesis, leading to peripheral blood cytopenias and an increased risk of progression to acute myeloid leukemia (AML). The disease encompasses various subtypes influenced by genetic mutations and chromosomal abnormalities, with specific lesions such as del(5q) and *TP53* mutations playing significant roles in disease pathology and prognosis [1, 2, 3].

*TP53*, the guardian of the genome, plays a crucial role in cell cycle regulation, response to DNA damage, and apoptosis. The p53 protein domains are composed of two transcriptional activation domains (TADs), including TAD1 and TAD2, a proline‐rich domain, a DNA‐binding domain (DBD), a tetramerization domain, and a negative regulatory domain [4]. *TP53* mutations are mainly concentrated in key functional domains, particularly the DBD. Most mutational events are missense mutations dispersed across more than half of the p53 protein, followed by nonsense mutations [5]. *TP53* accounts for 8%–10% of MDS, associated with complex karyotypes [6, 7], which are indicative of treatment response and poor prognosis. Recent classification of MDS with defining genetic abnormalities indicates that multi‐hit *TP53* mutations include two or more *TP53* mutations: a single mutation with evidence of loss of heterozygosity (LOH), or −17/del(17p), according to the fifth edition of the World Health Organization (WHO‐5) [8] and the 2022 International Consensus Classifications (ICC) [9].

Currently, the management of MDS is complicated by its biological diversity and the varying responses to therapeutic interventions [10, 11, 12]. Standard treatment approaches include hypomethylating agents and supportive care, but the presence of *TP53* mutations often correlates with resistance to conventional therapies and a dismal overall prognosis. Despite allogeneic hematopoietic stem cell transplantation (HSCT) is the only potentially curative option, the survival after HSCT remains generally poor, highlighting the urgent need for targeted treatment strategies tailored to the genetic profile of the disease [13, 14, 15]. Furthermore, with novel therapeutic agents targeting mutant *TP53* (e.g., eprenetapopt (APR‐246)) under active clinical investigation, improved risk stratification for patients with *TP53*‐mutated MDS has become increasingly urgent to better identify candidates for these emerging therapies and to interpret clinical trial outcomes [16]. Bernard et al. reported that the median overall survival (OS) was 8.7 months in the multi‐hit state, 2.5 years in the mono‐allelic state, and 3.5 years in the wild‐type state, based on a large cohort of 3148 MDS patients [17]. Previous studies have documented that *TP53* variant alle frequency (VAF) was correlated with MDS prognosis with different thresholds, including ≥ 20% from Montalban‐Bravo et al., ≥ 20% Montoro et al., and ≥ 22% from Tefferi et al., indicating higher VAF indicates a higher tumor burden in the patients and suggests poor prognosis [10, 14, 18, 19, 20]. Minimizing bone marrow (BM) blast cutoffs to discriminate *TP53*‐mutated MDS and AML was essential as both poor prognosis irrespective of blast percentage [9, 21, 22]. Symes et al. reported co‐occurring mutations play a key role in *TP53*‐mutated MDS [23]. Co‐occurring mutations in genes involved in key cellular pathways frequently modify disease phenotype and clinical outcomes in MDS. For instance, mutations in *DNMT3A* and *TET2* disrupt DNA methylation patterns, *ASXL1* mutations impair chromatin remodeling, and mutations in splicing factors such as *SF3B1* and *U2AF1* lead to aberrant RNA processing. The impact of these co‐mutations on the clinical behavior of *TP53*‐mutated MDS remains a critical unanswered question. Although the advancements in understanding the genetic underpinnings of MDS, there remains a notable gap in the literature regarding the mutation landscape in *TP53*‐mutated Asian MDS patients, and uncertainty about VAF correlations with specific clinical parameters beyond survival.

In this study, we employ next‐generation sequencing (NGS) to comprehensively analyze the mutational landscape of *TP53* along with other relevant genetic alterations. This analysis was performed on a large single‐center cohort of 1589 adult Chinese patients newly diagnosed with MDS. This approach enables us to capture a broader spectrum of mutations, providing insights into their distribution and potential clinical significance in the Asian population. By elucidating the relationship between genetic alterations and clinical outcomes, we aim to facilitate improved risk stratification and targeted therapies, ultimately enhancing patient care within the challenging clinical landscape affecting Asian patients.

## 2 Materials and Methods

### 2.1 Study Design

This was a retrospective, observational study conducted at The First Affiliated Hospital, Zhejiang University School of Medicine. A total of 1589 adult *de novo* MDS patients with BM sequencing samples at diagnosis were initially screened 01/07/2021 to 16/12/2024. Patients were included in this study if they met all of the following criteria: (1) A confirmed diagnosis of MDS according to WHO‐5 classification; (2) Availability of baseline BM samples for genetic analysis; (3) Presence of at least one *TP53* mutation detected by NGS at the time of initial diagnosis. No specific exclusion criteria were applied. 161 *TP53*‐mutated patients were enrolled in this study. The primary objective of this study was to characterize the spectrum and patterns of *TP53* mutations in a real‐world Asian MDS cohort. The secondary objective was to analyze the association between different *TP53* mutational states (e.g., single mutation vs. multiple mutations) and baseline clinicopathological features (e.g., demographics, peripheral blood parameters, BM characteristics, and cytogenetics). This study was approved by the Ethics Committee of The First Affiliated Hospital, Zhejiang University School of Medicine and all patients signed informed consent forms before enrollment.

### 2.2 Next‐Generation Sequencing Analysis

Patient BM DNA was extracted from the BM at diagnosis. Saliva samples were chosen to identify germline DNA, which was used to discriminate somatic mutations from germline variants in the final somatic mutation analysis. Gene library amplification was performed by a KAPA Hyper Prep Kit. The targeted 96‐gene sequencing panel was provided by Acornmed Biotechnology Co. Ltd. (Table S1). Multiplex libraries were sequenced via Illumina NovaSeq instrument. Raw variants were filtered based on the following criteria: an average effective sequencing depth on target per sample ≥ 800×; mapping quality ≥ 30 and base quality ≥ 30; and VAF ≥ 1% for single nucleotide variation (SNV) and insertion or deletion (InDel). Reads were aligned to the human genome (version: hg19) using Burrows‐Wheeler Alignment (BWA, version 0.7.12). The MarkDuplicates tool from Picard was utilized to identify PCR duplicates. BaseRecalibrator and IndelRealigner from Genome Analysis Toolkit (GATK, version 3.8) were used for recalibrating and realigning the BWA data, respectively. Mutect2 was employed to identify SNVs and InDels. The ANNOVAR software was used to annotate all variants, incorporating data from 1000G projects, COSMIC, SIFT, and Polyphen.

### 2.3 Statistical Analysis

Statistical tests were performed using SPSS (version 22.0) or R package (version 3.5.2). Continuous variables were compared using Mann–Whitney U or Kruskal–Wallis test. For comparisons of continuous variables between two independent groups (e.g., age > 60 vs. ≤ 60 years), the Mann–Whitney U test was used. For comparisons involving more than two independent groups (e.g., across International Prognositc Scoring System ‐ Molecular (IPSS‐M) risk categories), the Kruskal–Wallis test was employed, and no further pairwise comparisons were conducted for multiple testing, due to the limitation of the sample size. Categorical variables were compared using chi‐square or Fisher's exact test. Clinical cut‐off values were selected based on established criteria in the literature and consensus guidelines. For age, neutrophil, hemoglobin, karyotype, cytogenetic risk cut‐offs followed IPSS or revised IPSS (IPSS‐R) criteria. For BM blast percentage, we used the median value in our cohort study. A two‐sided *p* value < 0.05 was considered statistically significant.

## 3 Results

### 3.1 Patient Characteristics

The baseline demographic, hematologic, and prognostic features of 161 patients with *TP53*‐mutated MDS are presented in Table 1. The cohort had a median age of 69 years (range: 32–86 years), with a male predominance (94 males, 58.4%; 67 females, 41.6%). Hematologic parameters revealed a median white blood cell (WBC) count of 3.28 × 10^9^/L (range: 0.18–41.02 × 10^9^/L), a median hemoglobin level of 70.00 g/L (range: 44.00–161.00 g/L), and a median platelet count of 68.00 × 10^9^/L (range: 1.00–1051.00 × 10^9^/L). The median neutrophil count was 1.80 × 10^9^/L (range: 0.11–17.73 × 10^9^/L), while the median BM blast percentage was 5.50% (range: 0.50%–18.50%). Karyotypic analysis categorized 45 patients (28.0%) as having a good karyotype, 7 (4.3%) as intermediate, and 91 (56.5%) as poor. Cytogenetic risk stratification classified 3 patients (1.9%) as very good, 43 (26.7%) as good, 8 (5.0%) as intermediate, 9 (5.6%) as poor, and 80 (49.7%) as very poor. Using the IPSS, patients were stratified as low risk (12, 7.5%), intermediate‐1 (42, 26.1%), intermediate‐2 (65, 40.4%), or high risk (20, 12.4%). The IPSS‐R classified 5 patients (3.1%) as very low risk, 15 (9.3%) as low risk, 21 (13.0%) as intermediate risk, 25 (15.5%) as high risk, and 73 (45.3%) as very high risk. Using the published algorithm for calculating IPSS‐M [24], patients were categorized into six IPSS‐M risk groups: very low (4 patients, 2.5%), low (9 patients, 5.6%), moderate low (10 patients, 6.2%), moderate high (10 patients, 6.2%), high (21 patients, 13.0%), and very high (107 patients, 66.5%).

**TABLE 1:** Baseline characteristics of 161 *TP53*‐mutated MDS patients.

Baseline characteristics	*N* (%) or median (range)
Age, median (range) years	69 (32–86)
Gender	
Male, *N* (%)	94 (58.4%)
Female, *N* (%)	67 (41.6%)
White blood cells, median (range) × 10^9^/L	3.28 (0.18–41.02)
Hemoglobin, median (range) × g/L	70.00 (44.00–161.00)
Platelets, median (range) × 10^9^/L	68.00 (1.00–1051.00)
Neutrophils, median (range) × 10^9^/L	1.80 (0.11–17.73)
Bone marrow blasts, median (range) %	5.50 (0.50–18.50)
Karyotypes	
Good	45 (28.0%)
Intermediate	7 (4.3%)
Poor	91 (56.5%)
NA	18 (11.2%)
Cytogenetic risks	
Very good	3 (1.9%)
Good	43 (26.7%)
Intermediate	8 (5.0%)
Poor	9 (5.6%)
Very poor	80 (49.7%)
NA	18 (11.2%)
IPSS	
Low	12 (7.5%)
Intermediate 1	42 (26.1%)
Intermediate 2	65 (40.4%)
High	20 (12.4%)
NA	22 (13.7%)
IPSS‐R	
Very low	5 (3.1%)
Low	15 (9.3%)
Intermediate	21 (13.0%)
High	25 (15.5%)
Very high	73 (45.3%)
NA	22 (13.7%)
IPSS‐M	
Very low	4 (2.5%)
Low	9 (5.6%)
Moderate low	10 (6.2%)
Moderate high	10 (6.2%)
High	21 (13.0%)
Very High	107 (66.5%)

Abbreviations: IPSS, International Prognostic Scoring System; IPSS‐M, IPSS‐Molecular; IPSS‐R, revised IPSS; MDS, myelodysplastic neoplasms; NA, not applicable.

**FIGURE 1:**
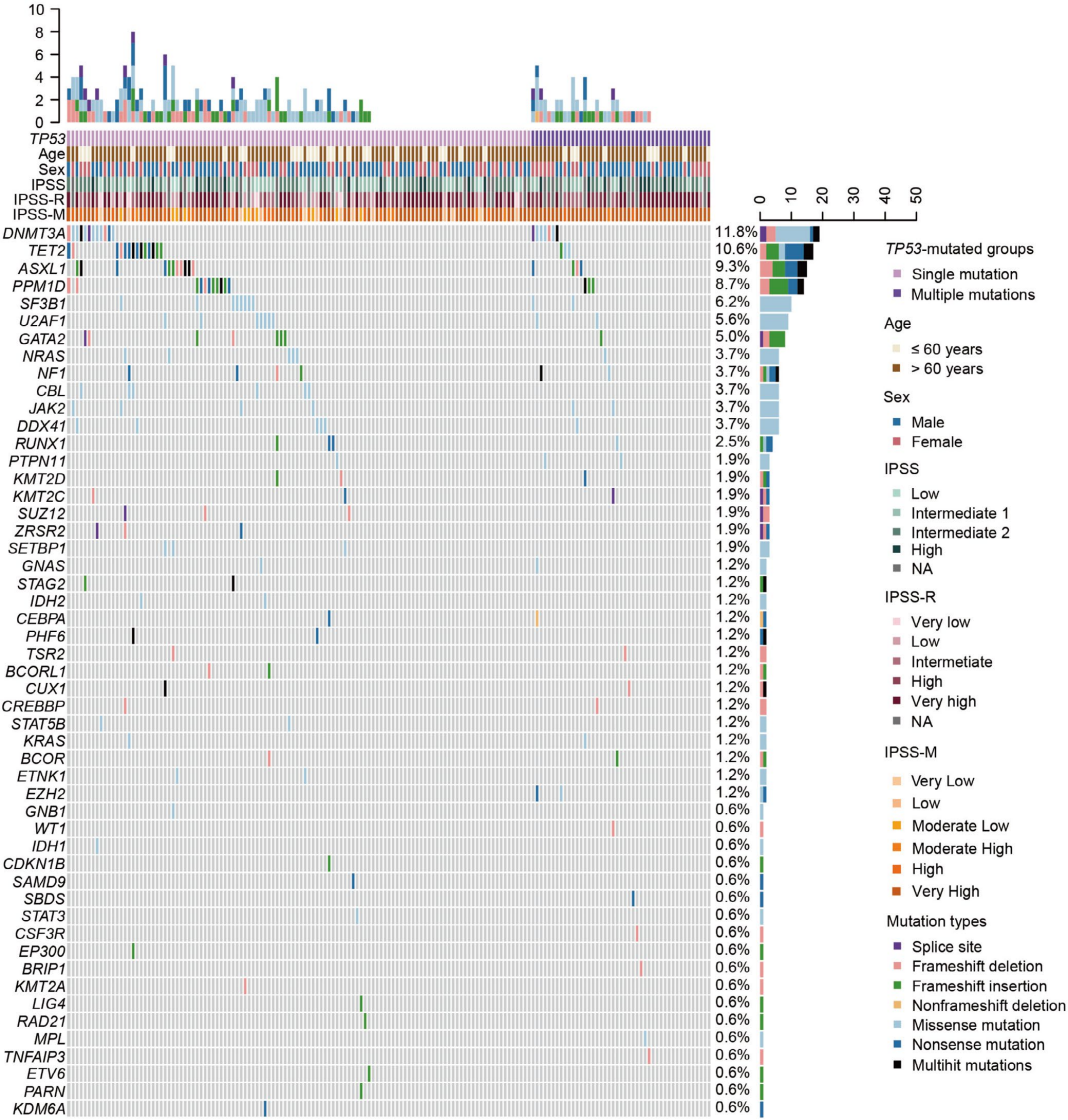
Distribution of gene mutations in 161 *TP53*‐mutated MDS patients. Heatmap showing the mutation details for each of the enrolled patients. Each column represents one patient, and each row corresponds to a mutation in the defined genes. IPSS, International Prognostic Scoring System; IPSS‐M, IPSS‐Molecular; IPSS‐R, revised IPSS; MDS, myelodysplastic neoplasms.

**FIGURE 2:**
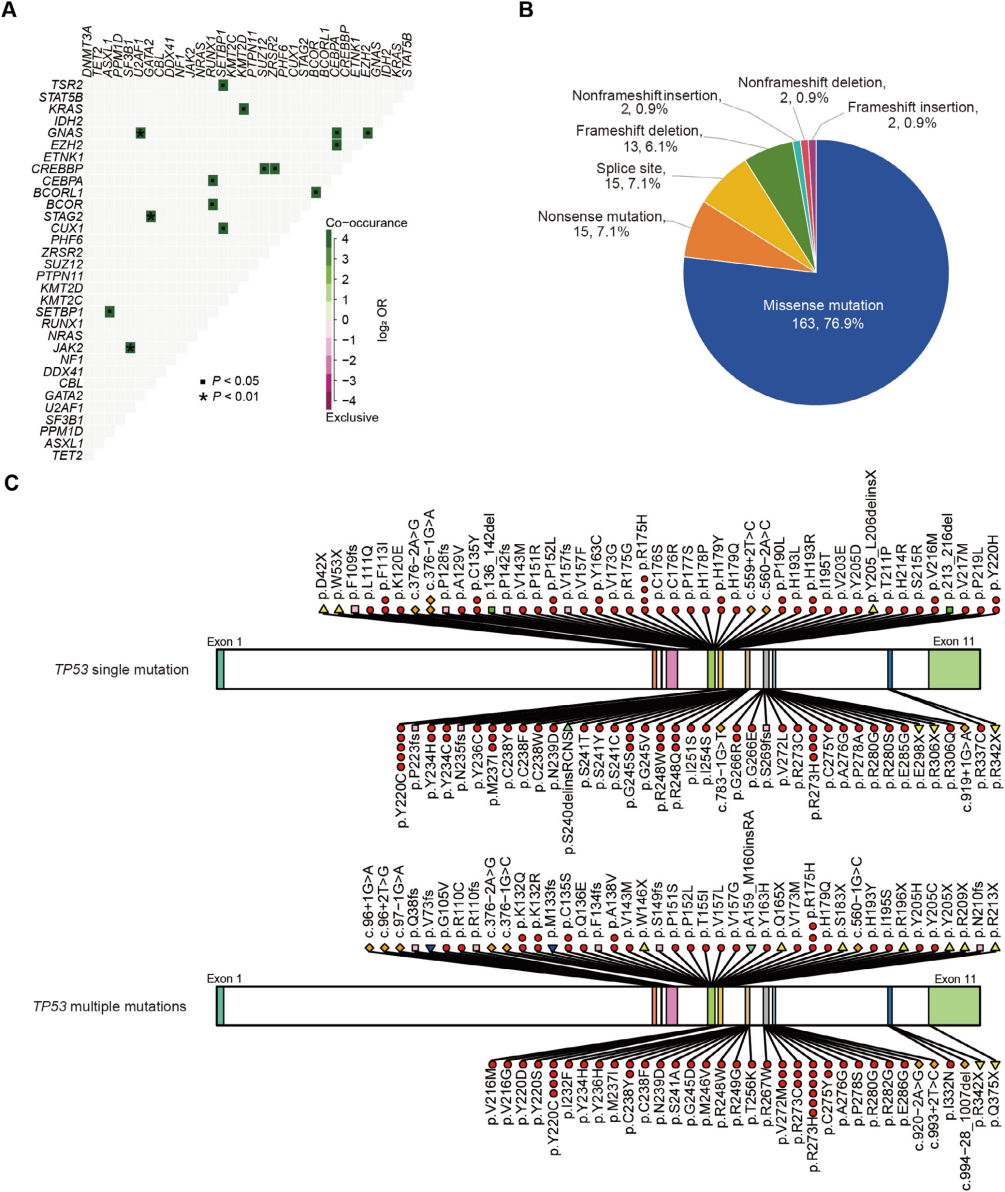
Correlation of detected mutations and *TP53*‐mutated characteristics in 161 *TP53*‐mutated MDS patients. (A) Pairwise associations among the genetic mutations. The co‐occurrence of each association is color coded, and the significance level is indicated by an asterisk or dot in each field. (B) Pie chart showing the fractions of *TP53* mutations according to the different mutation types. (C) Distribution of *TP53*‐mutated sites, according to single and multiple *TP53*‐mutated subgroups. MDS, myelodysplastic neoplasms.

Figure 1 summarizes the key features, including differentially clinical characteristics, and somatic mutation profiles identified in the cohort. Recurrent somatic mutations, except for mutated *TP53*, were identified across 51 genes. The most frequently mutated genes included *DNMT3A* (11.8%), *TET2* (10.6%), *ASXL1* (9.3%), *PPM1D* (8.7%), and *SF3B1* (6.2%). Other genes such as *U2AF1*, *GATA2*, *NRAS*, *NF1*, *CBL*, *JAK2*, and *DDX41* exhibited mutation frequencies of 4%–5%, while mutations in *RUNX1*, *PTPN11*, *KMT2D*, *KMT2C*, and so on, occurred at lower frequencies (1%–2%). Additionally, stratification by functional categories highlighted distinct mutation patterns. Epigenetics‐related genes demonstrated the highest mutation burden (41.6%), followed by signaling pathway genes (20.0%), and spliceosome genes (11.6%) (Figure S1A). Pairwise analysis of somatic mutations revealed distinct patterns of co‐occurrence and mutual exclusivity across 51 genes. Significant co‐occurrences were observed between mutated *RUNX1* and mutations in *CEBPA* and *BCOR*, as well as mutated *CREBBP* and mutations in *ZRSR2* and *SUZ12* (Figures 2A and S1B).

Based on the *TP53* mutation types, we discovered that missense mutations represented the predominant alteration type (76.9%), followed by nonsense mutations (7.1%), splice site variants (7.1%), and frameshift deletions (6.1%). Nonframeshift insertions, nonframeshift deletions, and frameshift insertions all accounted for 0.9% of mutations (Figure 2B). The classification of “multiple *TP53* mutations” in this specific context refers strictly to cases harboring two or more distinct *TP53* mutations. The distributions of *TP53*‐mutated sites, according to single and multiple *TP53*‐mutated subgroups, were shown in Figure 2C.

### 3.2 Comparisons of Mutated Genes in Different Clinical Subgroups

**FIGURE 3:**
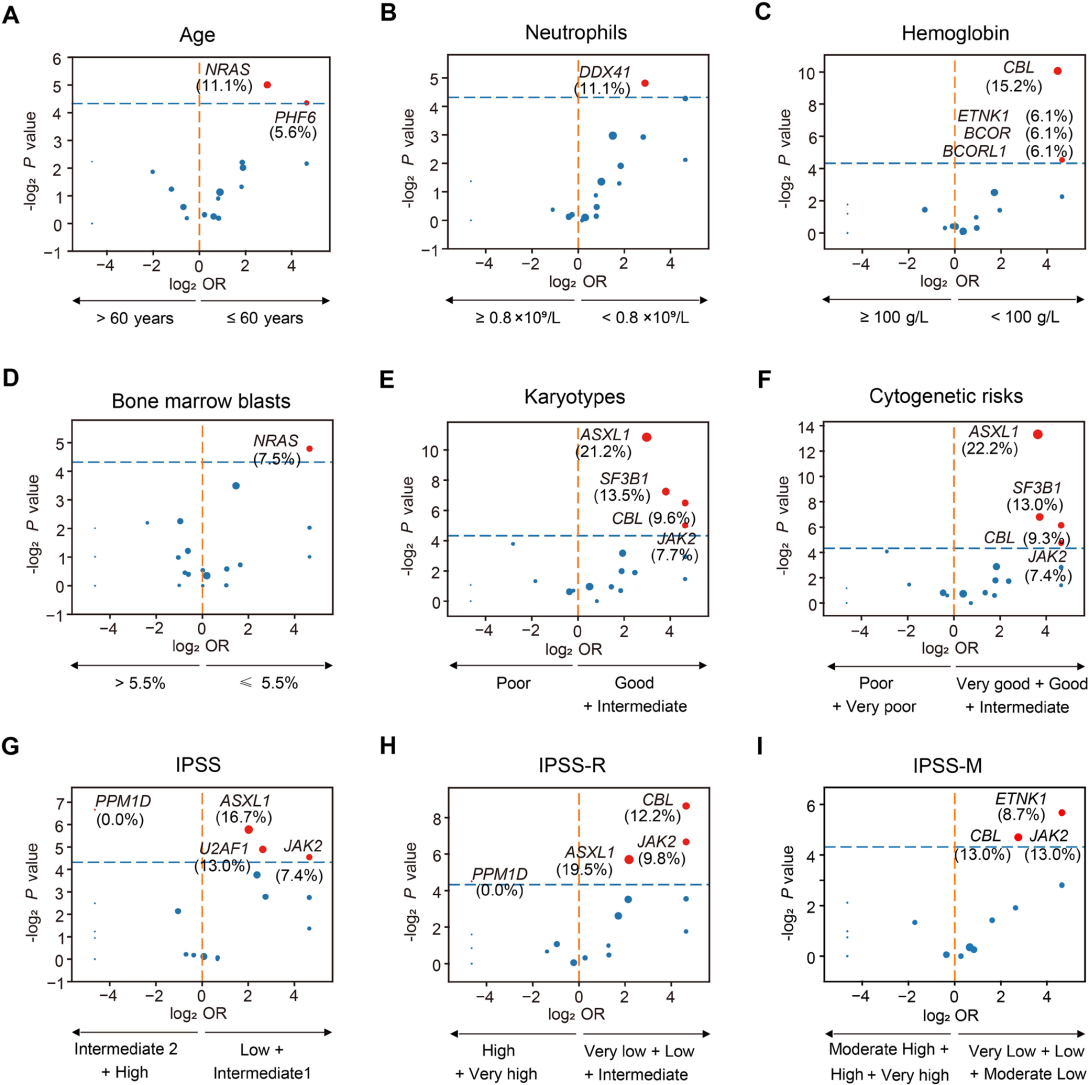
Association of mutated genes and clinical parameters, including age (A), neutrophils (B), hemoglobin (C), bone marrow blasts (D), karyotypes (E), cytogenetic risks (F), IPSS (G), IPSS‐R (H), and IPSS‐M (I). IPSS, International Prognostic Scoring System; IPSS‐M, IPSS‐Molecular; IPSS‐R, revised IPSS.

Subsequently, we compared the distribution of genetic mutations and their association with clinical features. We discovered that mutations in *NRAS* and *PHF6* were significantly associated with the younger age group (*p* = 0.031, *p* = 0.049, Figure 3A). *DDX41* mutations demonstrated a stronger correlation to lower neutrophil counts (*p* = 0.035, Figure 3B). Recurrent alterations in *CBL*, *BCORL1*, *BCOR*, and *ETNK1* exhibited effects in lower hemoglobin (all *p* < 0.050, Figure 3C). *NRAS* mutations were linked to lower BM blasts (*p* = 0.036, Figure 3D). The association of mutations in *ASXL1*, *SF3B1*, *CBL*, and *JAK2* were more prevalent in both patients with better karyotypes and cytogenetic risks (all *p* < 0.050, Figure 3E,F). Similarly, mutated *ASXL1*, *JAK2*, and *U2AF1* were more common in both patients with better IPSS and IPSS‐R, while *PPM1D* mutations were correlated to worse IPSS and IPSS‐R risk classifications (all *p* < 0.050, Figure 3G,H). We also discovered that mutated *ETNK1*, *CBL*, and *JAK2* were associated with better IPSS‐M risk category (all *p* < 0.050, Figure 3I). These findings highlight the heterogeneity of *TP53*‐mutated MDS and underscore the importance of genetic profiling.

### 3.3 Comparisons of *TP53* VAFs in Different Clinical Subgroups

**FIGURE 4:**
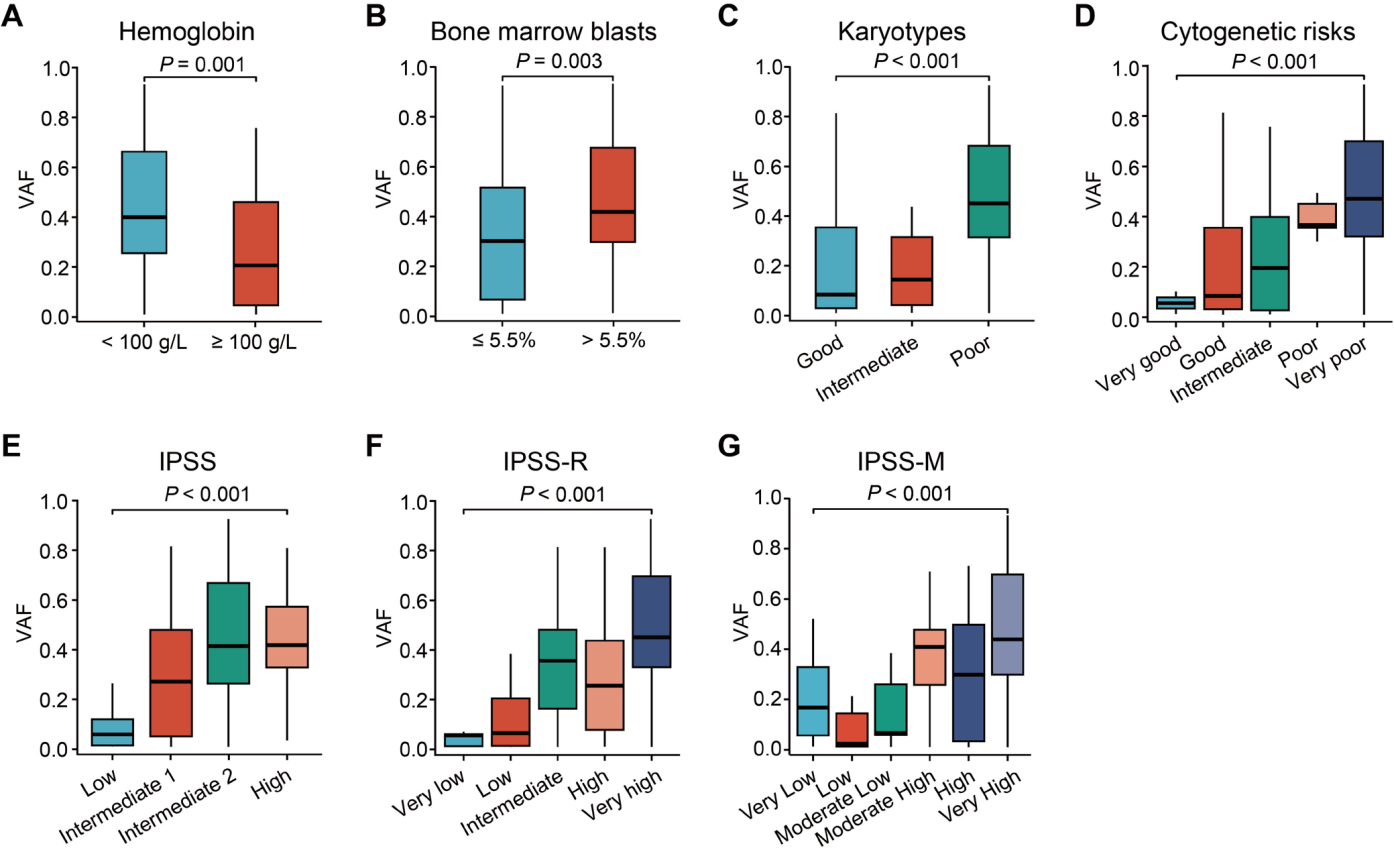
Association of *TP53* VAFs and clinical parameters, including hemoglobin (A), bone marrow blasts (B), karyotypes (C), cytogenetic risks (D), IPSS (E), IPSS‐R (F), and IPSS‐M (G). IPSS, International Prognostic Scoring System; IPSS‐M, IPSS‐Molecular; IPSS‐R, revised IPSS; VAF, variant allele frequency.

Furthermore, we drew the distribution of *TP53* VAFs in relation to various clinical features. Patients with hemoglobin < 100 g/L exhibited significantly higher VAF compared to those with hemoglobin ≥ 100 g/L (*p* = 0.001, Figure 4A), suggesting a correlation between anemia severity and clonal burden. Elevated BM blast counts (> 5.5%) were associated with increased VAF (*p* = 0.003, Figure 4B), indicative of higher mutational burden in advanced disease burden. *TP53* VAF progressively increased across karyotypes and cytogenetic risks, with the highest values observed in the two poorest subgroups (both *p* < 0.001, Figure 4C,D), reinforcing the prognostic relevance of cytogenetic complexity. IPSS, IPSS‐R, and IPSS‐M risk classifications showed strong associations with *TP53* VAFs (all *p* < 0.001, Figure 4E–G), aligning with mutational accumulation in advanced disease. The strong association of VAF with established prognostic indices (IPSS, IPSS‐R, IPSS‐M) provides preliminary support for its potential role as a biomarker for risk stratification in *TP53*‐mutated MDS, pending validation in prospective studies with direct survival outcomes.

### 3.4 Comparisons of Clinical Characteristics and Mutation Profiles in Single and Multiple *TP53* ‐Mutated Subgroups

According to the difference in the number of *TP53* mutations, the 161 *TP53*‐mutated MDS patients were divided into two subgroups, including *TP53* single mutation (*N* = 116) and *TP53* multiple mutation (*N* = 45) groups (Figure 1, Table 2). Patients with multiple *TP53* mutations were significantly older than those with single mutations (median age: 72 vs. 68 years, *p* = 0.006). No significant differences were observed in gender distribution (male: 55.6% vs. 59.5%; female: 44.4% vs. 40.5%, *p* = 0.650) or hematologic parameters, including WBC count (3.28 vs. 3.29 × 10^9^/L, *p* = 0.940), hemoglobin level (70.00 vs. 70.00 g/L; *p* = 0.850), platelet count (52.00 vs. 71.50 × 10^9^/L, *p* = 0.543), neutrophil count (1.89 vs. 1.63 × 10^9^/L, *p* = 0.741), and BM blast percentage (7.00% vs. 5.25%, *p* = 0.199). Karyotypic analysis revealed a higher proportion of poor karyotypes in the multiple mutation subgroup (*p* = 0.046), while cytogenetic risk stratification showed a trend toward more very poor risk in the multiple mutation group (*p* = 0.079). Prognostic classification by IPSS demonstrated a higher proportion of high‐risk patients in the multiple mutation subgroup (*p* = 0.039). IPSS‐R classification highlighted a significantly higher proportion of very high‐risk patients in the multiple mutation group (*p* = 0.039). Notably, no patients with multiple mutations were classified as low or very low risk under IPSS or IPSS‐R. Similarly, IPSS‐M classification demonstrated that higher risk patients were more prevalent among those with multiple *TP53* mutations (*p* = 0.001).

**TABLE 2:** Comparison of clinical characteristics of single and multiple *TP53*‐mutated subgroups in 161 MDS patients.

Baseline characteristics	Single mutation (*N* = 116)	Multiple mutations (*N* = 45)	*p*
Age, median (range) years	68 (32–86)	72 (36–85)	0.006
Gender			0.650
Male, *N* (%)	69 (59.5%)	25 (55.6%)	
Female, *N* (%)	47 (40.5%)	20 (44.4%)	
White blood cells, median (range) × 10^9^/L	3.29 (0.82–41.02)	3.28 (0.18–18.94)	0.940
Hemoglobin, median (range) × g/L	70.00 (44.00–161.00)	70.00 (44.00–159.00)	0.850
Platelets, median (range) × 10^9^/L	71.50 (1.00–1051.00)	52.00 (7.00–523.00)	0.543
Neutrophils, median (range) × 10^9^/L	1.63 (0.11–17.73)	1.89 (0.18–17.05)	0.741
Bone marrow blasts, median (range) %	5.25 (0.50–18.50)	7.00 (1.00–17.50)	0.199
Karyotypes			0.046
Good	38 (32.8%)	7 (15.6%)	
Intermediate	6 (5.2%)	1 (2.2%)	
Poor	60 (51.7%)	31 (68.9%)	
NA	12 (10.3%)	6 (13.3%)	
Cytogenetic risks			0.079
Very good	2 (1.7%)	1 (2.2%)	
Good	37 (31.9%)	6 (13.3%)	
Intermediate	7 (6.0%)	1 (2.2%)	
Poor	6 (5.2%)	3 (6.7%)	
Very poor	52 (44.8%)	28 (62.2%)	
NA	12 (10.3%)	6 (13.3%)	
IPSS			0.039
Low	12 (10.3%)	0 (0.0%)	
Intermediate 1	31 (26.7%)	11 (24.4%)	
Intermediate 2	48 (41.4%)	17 (37.8%)	
High	11 (9.5%)	9 (20.0%)	
NA	14 (12.1%)	8 (17.8%)	
IPSS‐R			0.039
Very low	5 (4.3%)	0 (0.0%)	
Low	15 (12.9%)	0 (0.0%)	
Intermediate	15 (12.9%)	6 (13.3%)	
High	16 (13.8%)	9 (20.0%)	
Very high	51 (44.0%)	22 (48.9%)	
NA	14 (12.1%)	8 (17.8%)	
IPSS‐M			0.001
Very low	4 (3.4%)	0 (0.0%)	
Low	9 (7.8%)	0 (0.0%)	
Moderate low	9 (7.8%)	0 (0.0%)	
Moderate high	10 (8.6%)	1 (2.2%)	
High	19 (16.4%)	2 (4.4%)	
Very High	65 (56.0%)	42 (93.3%)	

Abbreviations: IPSS, International Prognostic Scoring System; IPSS‐M, IPSS‐Molecular; IPSS‐R, revised IPSS; MDS, myelodysplastic neoplasms; NA, not applicable.

We also analyzed the differences in the distribution of mutated genes between these two groups. Unfortunately, we did not find a statistically different distribution of mutated genes. However, two genes, *EZH2* and *PTPN11*, tend to be in the multiple *TP53*‐mutated subgroup (*p* = 0.077, *p* = 0.190, Figure S2).

## 4 Discussion

We comprehensively analyzed the mutation spectrum in a cohort of 161 *TP53*‐mutated patients and assessed the correlation of these genetic alterations with clinical features and prognostic indicators via employing NGS technologies in an Asian cohort, and we make several key observations. First, the co‐mutational patterns were associated with clinical parameters. Second, *TP53* VAF revealed distinct biological subsets with potential prognostic implications. Third, we confirmed the clinical relevance of the multiple mutation framework that patients with multiple mutations exhibited significantly inferior prognostic indicators.

Our study provides significant innovations in the understanding of *TP53* mutations in MDS, particularly in highlighting the unique mutation profiles and their relations with clinical features. Patients with *TP53* mutations showed comparable median ages across different studies, with our cohort reporting a median age of 69 years, compared to 68 years in Kaur et al. [25], and 70 years in both Haase et al. [26] and Weinberg et al. [27], suggesting an age‐related tendency toward the development of *TP53*‐mutated clones. The *TP53* VAF provides critical insights into clonal architecture of patient diseases. Existing literature has established the association between *TP53* mutations, and poor response to standard therapies, and poor prognosis in MDS [18, 28, 29], especially for *TP53*‐mutated patients with VAF ≥ 20% [14] or ≥ 22% [30], consistent with our findings that higher VAF was correlated to unfavorable hematology, IPSS and IPSS‐R stratification index, which is crucial for diagnostics and tailoring treatment approaches. A high VAF signifies that the *TP53* mutation is a dominant, truncal event present in the vast majority of the malignant cells. This clone has achieved genomic dominance, defining the aggressive nature of the MDS and AML [10].

According to of WHO‐5 [8] as well as ICC 2022 [9], muti‐hit *TP53* mutation was recognized as a separate entity of myeloid neoplasms, suggesting the importance of molecular detection. Since the sequencing samples in our cohort were not subjected to LOH testing, the assessment of the *TP53* allelic state were not be fully defined. We note that without LOH data, the true prevalence of multi‐hit *TP53* cases (as per the strict WHO‐5/2022 ICC definition) may be underestimated in our cohort, as some single‐mutation cases could potentially be reclassified as multi‐hit if LOH were present. In our analysis, the classification of multiple *TP53* mutations refers strictly to patients harboring two or more distinct *TP53* mutations. Patients with multiple *TP53* mutations demonstrated more aggressive clinical features. This validates the biological relevance of the multi‐hit framework and its applicability to Asian MDS patients.

Our research uniquely identifies the specific distribution and common co‐occurring mutations within a defined cohort of *TP53*‐mutated MDS patients. Beyond *TP53* itself, our findings reveal that *DNMT3A*, *TET2*, and *ASXL1* mutations frequently coexist with *TP53* mutations, which underscores the complexity of MDS pathogenesis and suggests potential therapeutic targets other than *TP53* that have not been previously emphasized in other studies [31]. By elucidating these mutation patterns, our work fills critical gaps in knowledge and lays the groundwork for further personalized treatment strategies that could enhance patient outcomes.

These findings have direct implications for clinical practice. The clear distinction of both *TP53* quantities and VAFs should inform risk stratification beyond traditional IPSS/IPSS‐R scores. For clinicians, this research highlights the necessity of incorporating genetic testing into routine practice, which could guide decisions regarding more aggressive therapies such as HSCT or targeted therapies that address the specific genetic abnormalities observed in this patient population [32].

Unfortunately, there are some missing data for karyotype/cytogenetic risk (*n* = 18, 11.2%) and IPSS/IPSS‐R stratification (*n* = 22, 13.7%) in our study. This discrepancy arose because calculating these prognostic scores requires a complete set of cytogenetic data and specific components required for the scoring were also excluded from the IPSS/IPSS‐R analysis. The primary reason for the missing data is the detection technology that clear karyotype analysis results cannot be obtained for some samples. To assess the potential for selection bias, we compared the baseline characteristics (including age, gender, WBC, hemoglobin, platelets, neutrophils and BM blasts) the included and excluded patients according to karyotype/cytogenetic risk or IPSS/IPSS‐R category (Tables S2 and S3). We found no statistically significant differences in these key variables, suggesting that the missing data were likely missing at random and did not introduce a significant bias into our final results.

The limitations of this study primarily stem from the relatively small sample size, several missing karyotype data, and the absence of multi‐center clinical validation, and survival analysis, which may restrict the generalizability of our findings. Although our cohort of 161 *TP53*‐mutated MDS patients provides valuable insights into the mutation spectrum and clinical implications, the absence of longitudinal survival data precludes definitive assessment of prognostic impact, and a larger and more diverse population with complete standardized data is necessary to confirm these results and enhance the robustness of our conclusions. Although our cohort spans 2021–2024, the relatively short follow‐up period limits the statistical power and maturity required for robust survival analyses. However, active follow‐up is ongoing, and future studies with extended observation periods will incorporate comprehensive survival analyses, including Kaplan–Meier estimation with log‐rank comparisons and multivariable Cox proportional hazards modeling. These analyses will formally evaluate the prognostic value of VAF, multi‐hit allelic states, and specific co‐mutation patterns—variables we have now thoroughly characterized—in predicting survival. Such work will be critical to validate the risk stratification potential suggested by our current findings. Additionally, the retrospective nature of the study may introduce biases related to patient selection and data collection. Future research should aim to incorporate multi‐center approaches to validate our findings and explore the underlying biological mechanisms of *TP53* mutations in MDS.

## 5 Conclusion

In conclusion, this study elucidates the characteristics of *TP53* mutations and their association with other genetic alterations and clinical characters in Asian MDS patients. These findings support patient prognostic stratification and offer potential strategies for clinical management.

## Author Contributions

**Lixia Liu:** methodology, data curation, formal analysis, writing – original draft. **Hong Chen:** methodology. **Jiayue Qin:** conceptualization, methodology, investigation, writing – review and editing, supervision. **Wei Zhu:** data curation. **Yifei Wang:** data curation. **Yu Gao:** data curation. **Yanming Cheng:** methodology, formal analysis, data curation, writing – original draft. **Yudi Zhang:** conceptualization, methodology, investigation, writing – review and editing, supervision.

## Funding

The authors have nothing to report.

## Ethics Statement

This study was approved by the Ethics Committee of The First Affiliated Hospital, Zhejiang University School of Medicine ([2025B] IIT Ethics Approval No. 0810) and all patients signed informed consent forms for the publication.

## Consent

All patients signed informed consent forms for the publication.

## Conflicts of Interest

The authors declare no conflicts of interest.

## Supporting information


**Table S1:** Targeted 96‐gene sequencing panel.
**Table S2:** Comparison of clinical characteristics of included and excluded patients according to karyotype or cytogenetic risk category.
**Table S3:** Comparison of clinical characteristics of included and excluded patients according to IPSS or IPSS‐R category.
**Figure S1:** (A) Histogram showing the frequencies of the mutated genes in the represent different genetic pathways in 161 *TP53*‐mutated MDS patients. (B) Circos plot showing the detected mutations, corresponding to the relative frequency and pairwise co‐occurrence of mutations. MDS, myelodysplastic neoplasms.
**Figure S2:** The genetic feature of *TP53*‐mutated MDS. The frequencies of additional genetic alterations were analyzed in single and multiple *TP53*‐mutated MDS patients. MDS, myelodysplastic neoplasms.

## Data Availability

The data that support the findings of this study are available from the corresponding author upon reasonable request.
