# Supplementary material for: Genetic and Clinical Features in TP53‐Mutated Patients With Myelodysplastic Neoplasms: A Retrospective Study Based on Next‐Generation Sequencing Data
Source: Cancer Rep (Hoboken). 2026 May 29;9(6):e70584. doi: 10.1002/cnr2.70584 (PMC13239453; doi:10.1002/cnr2.70584)
Supplement: Supplementary file 1 — Table S1: Targeted 96‐gene sequencing panel. Table S2: Comparison of clinical characteristics of included and excluded patients according to karyotype or cytogenetic risk category. Table S3: Comparison of clinical characteristics of included and excluded patients according to IPSS or IPSS‐R category. Figure S1: (A) Histogram showing the frequencies of the mutated genes in the represent different genetic pathways in 161 TP53‐mutated MDS patients. (B) Circos plot showing the detected mutations, corresponding to the relative frequency and pairwise co‐occurrence of mutations. MDS, myelodysplastic neoplasms. Figure S2: The genetic feature of TP53‐mutated MDS. The frequencies of additional genetic alterations were analyzed in single and multiple TP53‐mutated MDS patients. MDS, myelodysplastic neoplasms. [file CNR2-9-e70584-s001.docx]

Supplementary information for

Genetic and clinical features in *TP53*-mutated patients with myelodysplastic neoplasms: a retrospective study based on next-generation sequencing data

**This file includes:**

Tables S1-S3

Figures S1-S2

**Table S1. Targeted 96-gene sequencing panel**

| *ANKRD26* | *CDKN1B* | *DNMT3A* | *GNAS* | *KRAS* | *POT1* | *SETBP1* | *STAT3* |
| --- | --- | --- | --- | --- | --- | --- | --- |
| *ABL1* | *CDKN2A* | *ELANE* | *HAX1* | *MLH1* | *PPM1D* | *SF1* | *STAT5B* |
| *ALK* | *CEBPA* | *EP300* | *IDH1* | *MPL* | *PRPF8* | *SF3B1* | *SUZ12* |
| *ASXL1* | *CECR2* | *ETNK1* | *IDH2* | *MSH6* | *PTEN* | *SH2B3* | *TERC* |
| *ATRX* | *CREBBP* | *ETV6* | *JAK2* | *MYC* | *PTPN11* | *SMC1A* | *TERT* |
| *BCOR* | *CSF3R* | *EZH2* | *JAK3* | *NF1* | *RAD21* | *SMCC3* | *TET2* |
| *BCORL1* | *CTCF* | *FAM46C* | *KDM5C* | *NPM1* | *RB1* | *SOS1* | *TNFAIP3* |
| *BLM* | *CUX1* | *FBXW7* | *KDM6A* | *NRAS* | *ROBO1* | *SRCAP* | *TP53* |
| *BRAF* | *DDX41* | *FLT3* | *KIT* | *NT5C2* | *ROBO2* | *SRP72* | *U2AF1* |
| *CALR* | *DHX15* | *GATA1* | *KMT2A* | *PDGFRB* | *ROBO3* | *SRSF2* | *USH2A* |
| *CBL* | *DKC1* | *GATA2* | *KMT2C* | *PHF6* | *RUNX1* | *STAG1* | *WT1* |
| *CD79A* | *DNAH2* | *GFI1* | *KMT2D* | *PIGA* | *SBDS* | *STAG2* | *ZRSR2* |

**Table S2. Comparison of clinical characteristics of included and excluded patients according to karyotype or cytogenetic risk category**

| Baseline characteristics | Included patients (N = 143) | Excluded patients (N = 18) | *P* |
| --- | --- | --- | --- |
| Age, median (range) years | 68 (32–86) | 72 (51–82) | 0.086 |
| Gender |  |  | 0.203 |
| Male, N (%) | 86 (60.1%) | 8 (44.4%) |  |
| Female, N (%) | 57 (39.9%) | 10 (55.6%) |  |
| White blood cells, median (range) ×10^9^/L | 3.30 (0.18–41.02) | 3.00 (0.84–15.00) | 0.779 |
| Hemoglobin, median (range) ×g/L | 69.00 (44.00–161.00) | 70.00 (47.00–159.00) | 0.659 |
| Platelets, median (range) ×10^9^/L | 68.00 (1.00–710.00) | 70.00 (2.00–1051.00) | 0.836 |
| Neutrophils, median (range) ×10^9^/L | 1.80 (0.11–17.73) | 1.87 (0.54–11.70) | 0.667 |
| Bone marrow blasts, median (range) % | 5.00 (0.50–18.50) | 7.50 (2.00–18.50) | 0.128 |

**Table S3. Comparison of clinical characteristics of included and excluded patients according to IPSS or IPSS-R category**

| Baseline characteristics | Included patients (N = 139) | Excluded patients (N = 22) | *P* |
| --- | --- | --- | --- |
| Age, median (range) years | 68 (32-86) | 71 (51-82) | 0.081 |
| Gender |  |  | 0.694 |
| Male, N (%) | 82 (59.0%) | 12 (54.5%) |  |
| Female, N (%) | 57 (41.0%) | 10 (45.5%) |  |
| White blood cells, median (range) ×10^9^/L | 3.30 (0.18-41.02) | 3.00 (0.84-15.00) | 0.779 |
| Hemoglobin, median (range) ×g/L | 69.00 (44.00-161.00) | 70.00 (47.00-159.00) | 0.659 |
| Platelets, median (range) ×10^9^/L | 68.00 (1.00-710.00) | 70.00 (2.00-1051.00) | 0.836 |
| Neutrophils, median (range) ×10^9^/L | 1.80 (0.11-17.73) | 1.87 (0.54-11.70) | 0.667 |
| Bone marrow blasts, median (range) % | 5.00 (0.50-18.50) | 7.00 (2.00-18.50) | 0.407 |

Abbreviations: IPSS, International Prognostic Scoring System; IPSS-R, revised IPSS.


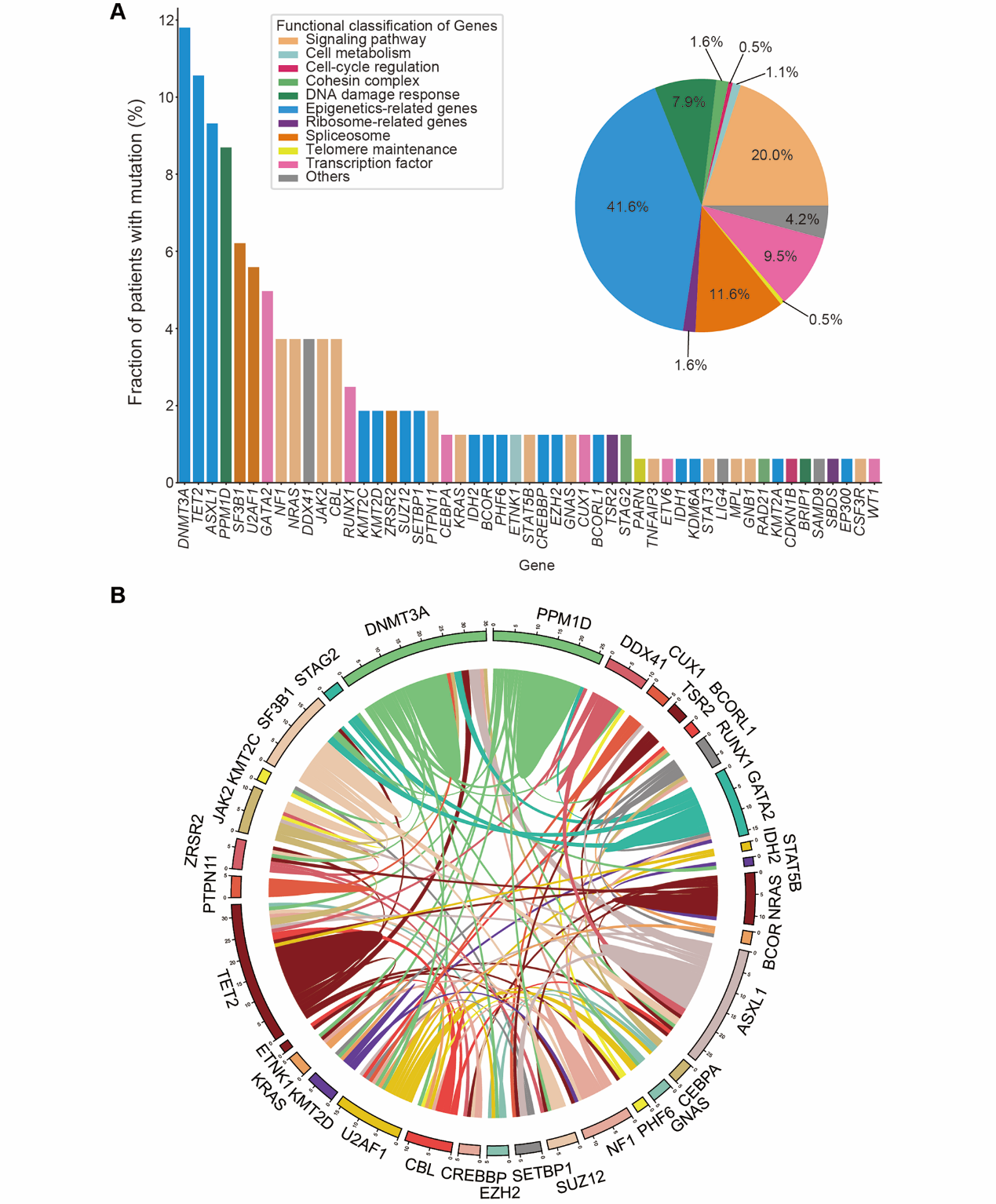


**Figure S1.** (A) Histogram showing the frequencies of the mutated genes in the represent different genetic pathways in 161 *TP53*-mutated MDS patients. (B) Circos plot showing the detected mutations, corresponding to the relative frequency and pairwise co-occurrence of mutations. MDS, myelodysplastic neoplasms.


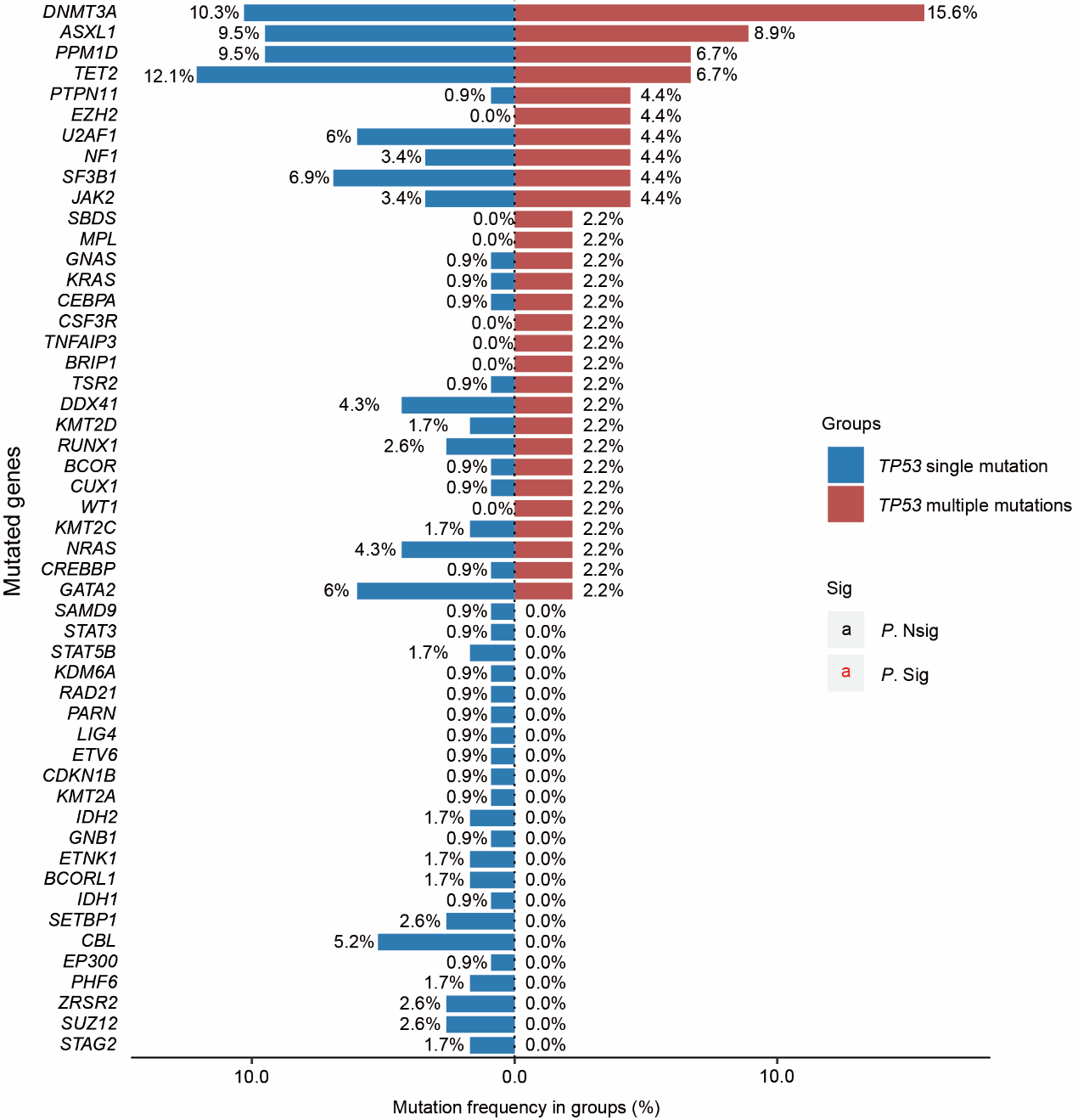


**Figure S2.** The genetic feature of *TP53*-mutated MDS. The frequencies of additional genetic alterations were analyzed in single and multiple *TP53*-mutated MDS patients. MDS, myelodysplastic neoplasms.
